# The Involvement of IL-17A in the Murine Response to Sub-Lethal Inhalational Infection with *Francisella tularensis*

**DOI:** 10.1371/journal.pone.0011176

**Published:** 2010-06-18

**Authors:** Gal Markel, Erez Bar-Haim, Eran Zahavy, Hila Cohen, Ofer Cohen, Avigdor Shafferman, Baruch Velan

**Affiliations:** 1 Department of Biochemistry and Molecular Genetics, Israel Institute of Biological Research, Ness Ziona, Israel; 2 Department of Infectious Diseases, Israel Institute of Biological Research, Ness Ziona, Israel; New York University, United States of America

## Abstract

**Background:**

*Francisella tularensis* is an intercellular bacterium often causing fatal disease when inhaled. Previous reports have underlined the role of cell-mediated immunity and IFNγ in the host response to *Francisella tularensis* infection.

**Methodology/Principal Findings:**

Here we provide evidence for the involvement of IL-17A in host defense to inhalational tularemia, using a mouse model of intranasal infection with the Live Vaccine Strain (LVS). We demonstrate the kinetics of IL-17A production in lavage fluids of infected lungs and identify the IL-17A-producing lymphocytes as pulmonary γδ and Th17 cells. The peak of IL-17A production appears early during sub-lethal infection, it precedes the peak of immune activation and the nadir of the disease, and then subsides subsequently. Exogenous airway administration of IL-17A or of IL-23 had a limited yet consistent effect of delaying the onset of death from a lethal dose of LVS, implying that IL-17A may be involved in restraining the infection. The protective role for IL-17A was directly demonstrated by *in vivo* neutralization of IL-17A. Administration of anti IL-17A antibodies concomitantly to a sub-lethal airway infection with 0.1×LD_50_ resulted in a fatal disease.

**Conclusion:**

In summary, these data characterize the involvement and underline the protective key role of the IL-17A axis in the lungs from inhalational tularemia.

## Introduction

*Francisella tularensis (Ft)*, the causative agent of tularemia, is a small gram-negative facultative intracellular bacterium, which can infect a broad spectrum of hosts. Infection of humans can be established by a variety of exposure routes, including infection via wounds, insect bites, ingestion, or inhalation [reviewed in 1]–[2]. The infectious dose required to cause human infection varies with the *Francisella* strain and route of entry [3]. *Ft* subspecies *tularensis* (type A subspecies) is a highly infectious and virulent pathogen that can cause a fulminant and often fatal disease by inhalational exposure to as few as 10 microorganisms. Therefore, type A *Ft* has been classified by the Center for Disease Control and Prevention (CDC) as a Category A bioterrorism agent [4]–[5].

Despite the disease severity and potential implications of inhalational tularemia, relatively little is known about the biology and interrelations of *Ft* with the host lung. *Ft* Live Vaccine Strain (LVS), an attenuated type B strain of *Ft*, causes a severe respiratory illness in mice that is commonly used to study inhalational tularemia [6]. Previous reports have shown that inhaled LVS infects preferentially airway macrophages and dendritic cells, as early as 1 h after infection, and continues to replicate within them [7]–[8]. Further, we have recently shown that dendritic cell trafficking is exploited by the bacterium for dissemination from the lungs to the draining lymph nodes [9]. Later in the course of infection, bacteria may also be found in considerable amounts in neutrophils [8].

As *Ft* is an intracellular pathogen and resides within cells of the respiratory tract, a role for pulmonary lymphocyte-mediated immune response is implicated. Lung-residing natural killer (NK) cells have been shown to become activated and to secrete IFNγ following intransal infection with LVS. Moreover, *in vivo* NK cell depletion studies have implied a protective role of NK cells [10]. However, the contribution of NK cells to protection from *Ft* remains unsolved as other reports suggested that NK cells may not exert an essential protective role [11]–[12]. A protective role for adaptive T cell mediated immunity against LVS infection was demonstrated in genetically immunodeficient mice, which died of overt infection one month after intradermal innoculation [13]. Mice depleted of CD4^+^ or CD8^+^ T cells, but not of both, [14]–[16], or mice with the corresponding knockout mutations [17], survived primary sublethal intradermal LVS infection, indicating that each of these subpopulations is capable of clearing primary infection with the pathogen. With regard to primary pulmonary infection, CD8^+^ T cell deficient mice were similarly susceptible to high dose intranasal LVS infection as wild type animals [11].

The *in vivo* contribution of IFNγ to the protection from LVS was clearly demonstrated in gamma interferon knockout (GKO) mice, which were highly susceptible to LVS infection via all routes [13], [18]. Additional experiments showed that IFNγ is required for early protection. It was reported that the presence of IFNγ during the first 2 days after sublethal intradermal infection ensures survival [19]. Neutralization of IFNγ by antibodies concomitantly to intradermal sublethal infection resulted in death of the mice (wild type, nude or SCID strains) within a week [13], [19]–[20]. These results concurred with earlier studies showing that IFNγ contributes to control of intracellular growth of *Ft* in macrophages [21]. Noteworthy, IFNγ has an important role in host protection from a diversity of intracellular bacteria, including *L. monocytogenes, M. tuberculosis, M. avium, S. typhimurium and C. trachomatis* [reviewed in 22].

Nevertheless, it was previously reported that LVS-infected IL-12p35^−/−^ mice, which fail to mount a robust IFNγ response, were still able to clear intradermal LVS infection [23]. Further, recent data show that *in vivo* primed T cells derived from LVS-infected lungs control intramacrophage LVS growth *in vitro*, mainly through IFNγ-independent mechanisms [24]. While it was shown that TNFα contributes to immune response against LVS as well [20], [25], additional T cell derived effectors, such as the IL-17A, may be involved in the response to LVS.

IL-17A is an early cytokine with pleiotropic effects, involved mainly in triggering inflammatory responses, such as inflammatory cytokines (e.g. IL-6, TNFα), chemokines and cell adhesion molecules, which collectively induce inflammation and neutrophil recruitment [reviewed in 26]. As a consequence, IL-17A is involved in host protection against a broad spectrum of pathogens, including intracellular bacteria [reviewed in 26]–[28]. The major lymphocyte sources for IL-17A are the unique Th17 cells, which are developed due to the function of the IL-23 cytokine, as well as the γδ T cells [reviewed in 29]. Both IL-17A-producing T cell subpopulations have been implicated in host resistance to various intracellular bacteria [27]–[29]. However, the involvement of the IL-17A axis in inhalational *Ft* infection has been limitedly studied so far. Main evidence includes demonstration of the presence of Th17 cells in lungs of mice intranasally infected with LVS [30], and that *in vitro* exposure of human peripheral blood monocytes to *F. novicida* induce the production of IL-23 [31].

Here we provide direct and substantial evidence for the involvement of IL-17A in host defense to inhalational LVS infection. We identified the IL-17A-producing pulmonary lymphocytes and demonstrated the kinetics of their appearance, as well as of the IL-17A cytokine, in the lungs lavage fluids in response to inhalational infection. The protective role *in vivo* of IL-17A is demonstrated by *in vivo* neutralization and exogenous airway administration of cytokines. These results underline the effect of IL-17A on overall host response and survival from inhalational LVS infection.

## Results

### Pulmonary lymphocytes are activated following lethal intranasal infection with 100×LD_50_ LVS

**Figure 1:**
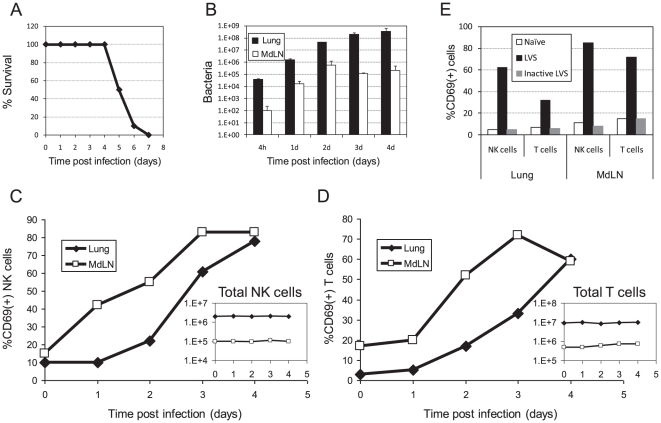
The effect of lethal intranasal infection with 100×LD_50_ LVS on activation and recruitment kinetics of lymphocytes in the respiratory system. (A) Survival of 60 C57BL/6 mice in after intranasal infection with 100×LD_50_ LVS (Accumulation of six independent experiments with 10 mice in each experiment); (B) Live bacterial counts in lungs (dark bars) and mediastinal lymph nodes (MdLN) (light bars) at the indicated time points after infection. The average count in three animals is shown in each time point. (C) CD69 expression analysis on gated NK cells (NK1.1^+^CD3^−^ cells) in the lungs (closed diamonds) and MdLN (open squares) at the indicated time points after infection (D) CD69 expression analysis on gated T cells (NK1.1^−^CD3^+^ cells). The respective *insets* in both (C) and (D) show the total numbers of NK or T cells; Cells were pooled from three animals at each time point and analyzed; (E) CD69 expression on gated NK and T cells in the lungs or MdLN three days following instillation of PBS (white bars), 100×LD_50_ live LVS (black bars) or equivalent dose of inactive LVS (gray bars). Cells were analyzed from three pooled animals. Panels B-E depict a representative experiments out of three independent experiments performed. Each time point included three animals.

To evaluate the effects of inhalational tulatermia on the function of pulmonary lymphocytes, we have used a model of lethal intranasal infection of mice by LVS [9]. C57BL/6 mice were infected with 10^5^ CFU of LVS, the equivalent of 100×LD_50_, which results in the death of all mice by day 5–6 post infection (Figure 1A). Live bacteria can be detected in the mediastinal lymph node (MdLN) already by 4 h, with a dramatic 1000-fold increase during the first two days [9]. In the lungs, a dramatic bacterial multiplication was similarly observed (Figure 1B). As demonstrated previously [9], [32], over the last two days, bacterial numbers in the lung were further increased by additional 10-fold, while remained constant in the MdLN (Figure 1B).

Lymphocytes involved in cell-mediated immunity, chiefly NK cells and T cells, were analyzed concomitantly using multi-colored staining in flow cytometry. NK cells were defined as CD3-negative/NK1.1-positive cells. T cells were defined as CD3-positive/NK1.1-negative cells. NK-T cells (CD3-positive/NK1.1-positive cells) were identified in small fractions and were excluded (data not shown). No significant alterations in the total NK cell counts were observed in either lungs or MdLN along the course of infection (Figure 1C, *inset*). However, a rapid upregulation of the activation marker CD69 was observed on the NK cells in the MdLN already after one day, and in the lungs starting from day 2. By days 3–4, the vast majority of NK cells in the lung and MdLN displayed an activated phenotype (Figure 1C), even though the total NK cell count remained unchanged.

As in the case of NK cells, total T cell count in MdLN and lungs was essentially not effected by LVS infection (Figure 1D, *inset*). A substantial increase in the percentage of activated T cells was observed, which lagged after NK cell activation by at least one day in both lungs and MdLN (Figure 1D). This concurs with the innate properties of NK cells. By day 4, around 60% of the T cells were activated.

Multi colored analysis with antibodies directed against CD3, NK1.1, αβTCR and CD69 yielded similar results (Supplementary Figure S1). Analysis of the late activation marker CD25 yielded similar patterns, but expectedly to a lesser extent (data not shown). Similar results were observed in another mouse strain, Balb/c (data not shown). In addition, it should be noted that lymphocytes were activated only by the presence of live bacteria. This was demonstrated by instillation of inactivated LVS in a dose-equivalent to 10^8^ CFU. This high dose, which is equivalent to the dose present in the lung at the peak of infection with 10^5^ CFU live bacteria (see Figure 1B), failed to induce CD69 expression on NK or T cells, in either the lungs or MdLN (Figure 1E).

Taken together, these results indicate that intranasal LVS infection triggers a rather rapid activation of lymphocytes involved in cell-mediated immunity. The increase in activation level coincides with increase of bacterial load in the infected organ and reaches maximal levels shortly before mice succumb to infection. This suggests that the host responds appropriately to the airway infection by local lymphocyte activation, but the limited activation kinetics fails to control the fatal outcomes of an infection, characterized by a high bacterial burden.

### The effect of sub-lethal intranasal infection with 0.1×LD_50_ LVS on activation and recruitment kinetics of pulmonary lymphocytes

**Figure 2:**
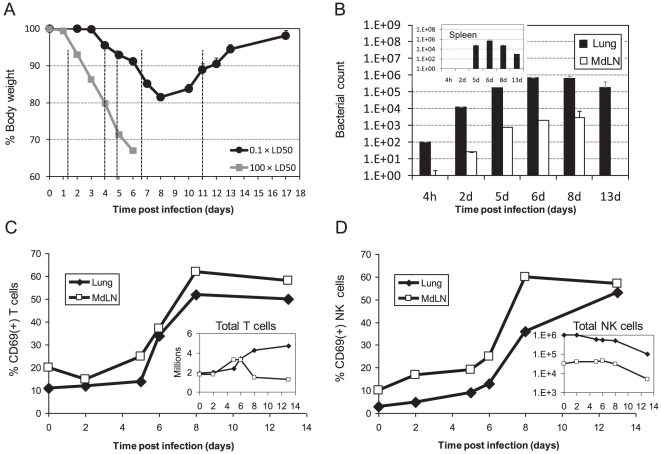
The effect of sub-lethal intranasal infection with 0.1×LD_50_ LVS on activation and recruitment kinetics of lymphocytes in the respiratory system. (A) The mean weight of 60 animals in several independent experiments following intranasal infection with 0.1×LD_50_ LVS (black circles) or with 100×LD_50_ LVS (gray squares) is presented (Accumulation of six independent experiments with 10 mice in each experiment). Dashed vertical lines mark the time points selected for bacterial immunological analyses; (B) Live bacterial counts in the lungs (dark bars) and mediastinal lymph nodes (MdLN) (light bars) at the indicated time points after infection. *Inset* shows the concomitant live bacterial count in the spleen. The average counts out of three animals is shown in each time point; CD69 expression analysis on (C) gated T cells (NK1.1^−^CD3^+^ cells) and (D) gated NK cells (NK1.1^+^CD3^−^ cells) in the lungs (closed diamonds) and MdLN (open squares) at the indicated time points after infection. The respective *insets* show the total numbers of NK or T cells. Cells were analyzed from three pooled animals at each time point. Panels B–D depicts a representative experiment out of three independent experiments performed. Each time point included three animals.

In an attempt to search for a possible interrelationship between lymphocyte function and recovery from infection, we have examined a model using a lower bacterial infection burden. Infection with 0.1×LD_50_ of LVS caused a pronounced yet non-fatal illness in almost all mice, and could be characterized by several consecutive phases: a subclinical phase (first 2–3 days post infection), an initial illness evident by a mild decrease in total weight (days 4–5), a full blown disease evident by pronounced weight decrease and lethargy (days 6–10), which is followed by a convalescence phase (day 11, and on) (Figure 2A). These disease manifestations concur with previous reports [32], and are in contrast to the rapid and consistent decrease in body weight of mice infected with lethal dose of 100×LD_50_ (Figure 2A).

Bacterial dissemination and lymphocyte activation were concomitantly analyzed at selected time points. During the subclinical phase (day 2), bacterial counts increased by 100-fold in the lungs, and could be just barely detected in the MdLN (Figure 2B). During the peak of disease (days 6–8), bacterial counts further increased by additional ∼100-fold to a maximum of ∼10^6^ CFU and ∼10^3^ CFU per a pair of lungs and MdLN, respectively (Figure 2B). It should be noted that the systemic bacterial distribution, as evident by bacterial counts in the spleen, was also maximal by day 6, but began to subside by day 8 and was already lower by 100-fold by day 13 (Figure 2B, *inset*).

Transition from one stage of the disease to another coincided with a notable change in the dynamics of pulmonary T cell numbers and state of activation. T cell representation was not effected during the first days of the subclinical disease. Yet, the development of clinically evident disease coincided with an increase in T cell numbers and in their activation as manifested by CD69 display (Figure 2C). The T cell numbers and activation status reached their peak with the peak of disease and remained constant during convalescence. In the MdLN, however, the observed increase in T cell numbers was transient and returned to baseline during convalescence (Figure 2C, *inset*).

The dynamics of pulmonary NK representation in (Figure 2D) differed substantially from that observed for T cells. The number of NK cells decreased gradually along the course of the sub-lethal infection (unlike the case in lethal infection), resulting in a 10-fold decrease by day 13. Notably, multi colored analysis with antibodies directed against CD3, NK1.1, αβTCR and CD69 yielded similar results (Supplementary Figure S2). One should note that infection triggers activation of the pulmonary NK cells, but given the decrease in total NK cell number, the net number of activated NK cells remains essentially unchanged along the infection process.

### Cytokine production kinetics by pulmonary NK and T cells during LVS infection

The temporal co-incidence between lymphocyte activation and the leveling-off in the pulmonary bacterial burden (Figure 2) could suggest that lymphocytes may exert defensive mechanisms contributing to bacterial clearance and eventually recovery. To further characterize the nature of the lymphocyte response, cytokine expression profile by pulmonary lymphocytes of infected mice was monitored.

**Figure 3:**
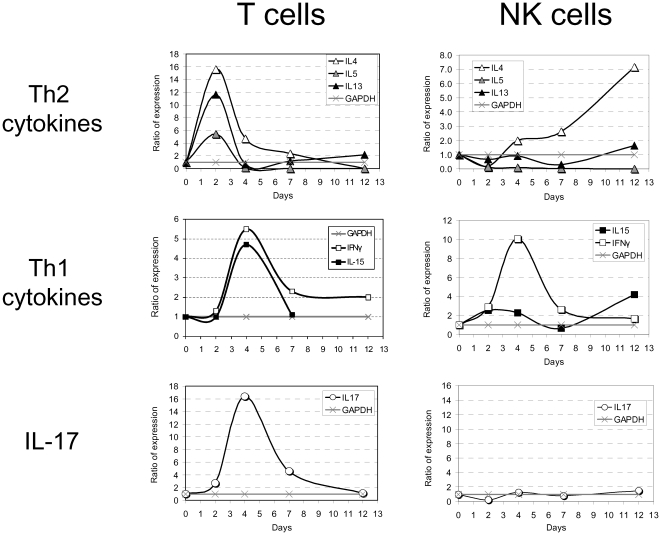
Cytokine expression profile in purified pulmonary T and NK cells following infection with 0.1×LD_50_. Figure shows the expression ratio (see below) of cytokines categorized into three groups (indicated on the left) in two main purified pulmonary lymphocyte subpopulations, NK and T cells, at indicated time points following infection with 0.1×LD_50_. The quantity of cytokine transcripts was determined as described in Materials and Methods.

T and NK cells were sorted out using flow cytometry from the lungs of naïve mice (day 0), or from LVS-infected mice following infection with 0.1×LD_50_ at days 2, 4, 7 and 12. Total RNA was extracted from the sorted cells and the expression of different cytokines was quantified by Real Time PCR. Unexpectedly, an early and strong, yet transient, induction of expression of the Th2 cytokines IL-4, IL-5 and IL-13 was measured in pulmonary T cells by day 2, before clinical manifestations became noticeable (Figure 3). Concurrently, pulmonary NK cells exhibited a gradual increase in the expression of IL-4 transcripts, but not of IL-5 or IL-13. As expected, and in line with previous reports [16], the Th1-biasing cytokine IFNγ was clearly induced in pulmonary T cells by day 4. This was followed by notable decline, yet IFNγ expression remained mildly enhanced up to day 12. Similarly, pulmonary NK cells exhibited an early induction of IFNγ already by day 2, a robust peak by day 4 and a subsequent downregulation. IL-15 was induced only in pulmonary T cells.

Most notably, an early and robust induction of IL-17A was observed in pulmonary T cells, but not in pulmonary NK cells (Figure 3). IL-17A induction in T cells is characterized by a sharp peak on day 4 which subsided subsequently. Interestingly, the IL-17A peak corresponded temporally with the development clinical symptoms, while its downregulation corresponded with restraining of the disease and eventual recovery.

### Production of IL-17A in the lungs following intranasal infection with LVS

**Figure 4 pone-0011176-g004:**
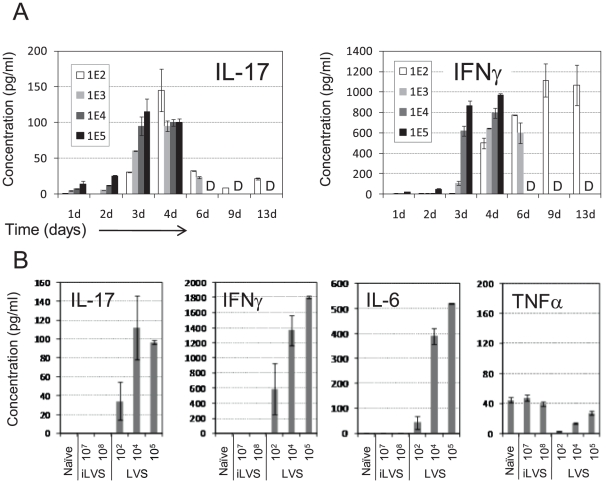
Accumulation of cytokines in the respiratory tracts following intranasal infection with LVS. (A) IL-17A and IFNγ concentrations in BALFs of mice infected with various infective doses at the indicated time points. Each of the concentration values represents the mean of 2–3 BALFs collected from individual animals. The letter “D” indicates that the mice infected with the corresponding infective doses could not be measured due to death of the animals. (B) The mean concentration of the indicated cytokines three days post instillation of PBS (naïve), inactive (iLVS) or live (LVS) bacteria. Each of the concentration values represents the mean of 2–3 individual BALFs. Representative experiment out of three performed is shown in panels A and B.

To monitor the actual levels of cytokine synthesis, the accumulation of prototype cytokines was measured in bronchoalveolar lavage fluids (BALF) of infected mice. Mice were instilled with four different infective doses ranging from 0.1×LD_50_ (10^2^ CFU) to 100×LD_50_ (10^5^ CFU). BALFs were harvested at different time points and the concentrations of IL-17A and IFNγ were determined by ELISA (Figure 4A). In agreement with previous reports [10], [16], a clear dose-dependent induction of IFNγ was observed (Figure 4A, *right*). Higher infective doses resulted in an earlier induction of IFNγ, as well as in increased production of IFNγ. Intranasal infection with LVS, which induces IL-17A expression in pulmonary lymphocytes (Figure 3), entailed accumulation of IL-17A in the BALF (Figure 4A *left*) that reached its peak in days 3–4. In contrast to the accumulation profile of IFNγ, IL-17A production displayed only a partial dose-dependent induction pattern. Although higher infective doses resulted in an earlier induction, there were no apparent differences in time of induction or in strength of induced response between infection with 10×LD_50_ and 100×LD_50_ (Figure 4A, *left*). Moreover, when enough time was allowed (day 4 of infection) the amounts of IL-17A in BALFs of mice infected with 100 CFU was found to be equivalent to that induced by infection with 10^5^ CFU.

Sub-lethal infection (0.1×LD_50_) allowed the monitoring of cytokine accumulation over a prolonged course of time. The amounts of IFNγ in the BALF increased gradually and remained very high even through the convalescence period (Figure 4A). This concurs with the observation depicted in Figure 3, showing that the mRNA for IFNγ remained above basal levels in both T and NK cells, even during the convalescence period. In contrast, IL-17A production in sub-lethal infection reached a peak on day 4 that rapidly subsided, concurring with the observed transient mRNA induction (Figure 3). Inactivated LVS in high dose-equivalents did not induce, within three days, the production in the lungs of IL-17A, nor of IFNγ or other inflammatory cytokines such as IL-6 or TNFα (Figure 4B). This is in accordance with the results depicted in Figure 1E, where lymphocyte activation was shown to depend on the viability of the instilled bacterium.

Similar to IFNγ, IL-6 also displayed a dose-dependent induction pattern, while TNFα was not induced even in very high infective doses (Figure 4B). It was previously reported that *F. tularensis* may actively inhibit the production of TNFα by macrophages and even compete with LPS [33].

Taken together, these observations provide evidence that IL-17A is indeed produced in the lungs in response to inhalational LVS infection, and in accordance with other observations [30], [34]–[35]. The inflammatory cytokines IFNγ and IL-6 are concomitantly produced in the lungs following infection as well. As opposed to IFNγ and IL-6, the production of IL-17A could not be further induced by infection doses beyond 10×LD_50_.

### Intranasal LVS infection triggers a robust production of IL-17A by specific pulmonary T cell subpopulations

**Figure 5 pone-0011176-g005:**
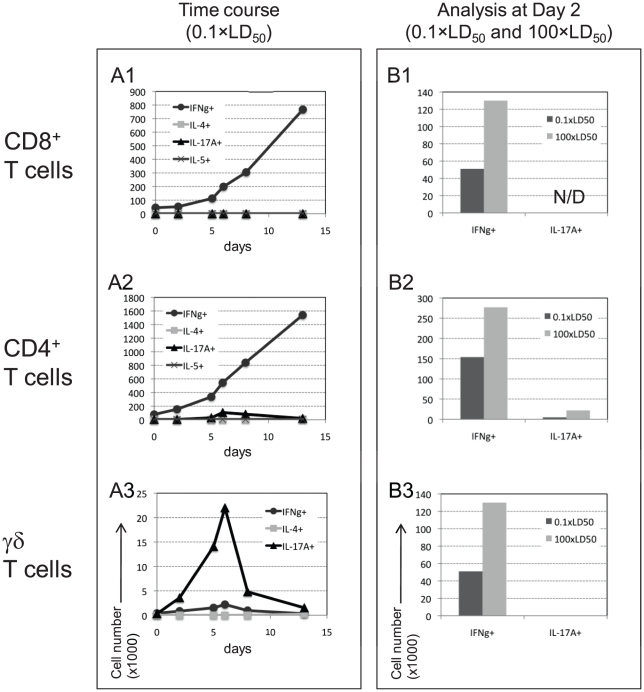
Expression of cytokines by specific pulmonary T cell subpopulations following intranasal infection. Three T cell subpopulations (indicated on the left) were examined for expression of selected cytokines. Panel (A1–A3) show the number of positive cells, stained intracellularly for the production of the indicated cytokines at the indicated time points following infection with 0.1×LD_50_ LVS; (B1–B3) compare the number of cells expressing IFNγ or IL-17A at day 2 post infection with 0.1×LD_50_ (black bars) or with 100×LD_50_ (gray bars). *N/D* stands for *Not Detected*. Cells were analyzed from three pooled animals at each time point. Figure depicts a representative experiment out of three experiments performed.

In order to evaluate in situ stimulation of pulmonary lymphocytes following intranasal infection, cells were collected at different time points post infection with 0.1×LD_50_ LVS. Production of the selected cytokines by different pulmonary T-cell lymphocyte subpopulations was characterized using intracellular staining (No additional *in vitro* stimulus was exerted). Three main pulmonary T cell subpopulations were analyzed: CD8^+^, CD4^+^ and γδ T cells. CD8^+^ T as well as CD4^+^ T cells displayed a strong, time-dependent increase in the fraction of IFNγ-producing cells (Figure 5, A1-A2), while γδ T cells failed to express IFNγ. This is consistent with previous reports [16].

As expected, IL-4, IL-5 were not expressed by CD8^+^ T cells at all (Figure 5, A1), but contrary to the expectation (Figure 3), these cytokines were not expressed by CD4^+^ T cells as well (Figure 5, A2). Thus, the transient infection-induced enhancement in mRNA of Th2 cytokines that we have observed in the pooled T-cell population (Figure 3) does not in fact translate into the protein level. One cannot exclude, however, the possibility of limited detection of the technique employed in this study.

LVS infection resulted in a distinct increase in IL-17A^+^ producing cells both in CD4^+^ T and γδ T cell populations. Among CD4^+^ T cells, IL-17A-producing cells reached the peak on day 6 post-infection (Figure 5, A2). This subpopulation was identified as Th17 cells, as they were negative for IFNγ or IL-4 (data not shown). These results concur with previous reports [30], [34]. These Th17 cells appeared on day 5, peaked at day 6 (3.2% of total population, data not shown) and subsided gradually (Figure 5, A2). Remarkably, a robust and early induction of IL-17A production by γδ T cells was observed as early as day 2-post infection, and reached the peak by day 6 (40% of the γδ T cells, data not shown) (Figure 5, A3).

The production of IL-17A by both Th17 and γδ T cells, exhibited a dose dependent pattern. Infection with 100×LD_50_ significantly enhanced IL-17A production as compared to infection with 0.1×LD_50_, when measured on day 2 (Figure 5, B2-B3). CD8^+^ T cells failed to produce IL-17A even at the high infection dose (Figure 5, B1). A dose dependent pattern was also observed with production of IFNγ by CD8^+^ T and CD4^+^ T cells, but not by γδ T cells (Figure 5, B1-B3).

### The contribution of various pulmonary T-cell subpopulations to production of IL-17A

**Figure 6:**
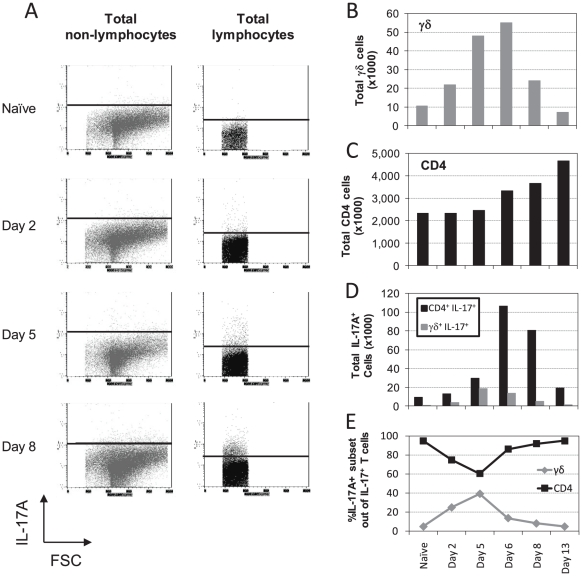
Kinetics of IL-17A producing pulmonary T cells following intranasal infection. (A) shows the IL-17A staining in total pulmonary non-lymphocyte cells (*left*) or lymphocytes (*right*), at the indicated time points. The total number of pulmonary γδ (B) or CD4^+^ (C) T cells at indicated time points post intranasal infection with 0.1×LD_50_ LVS; (D) Total number of IL-17A^+^ pulmonary γδ or (gray bars) or CD4^+^ (black bars) T cells at indicated time points post intranasal infection. (E) Percentage of IL-17A-positive T cell subset out of the total IL-17A-positive T cells. Cells were analyzed from three pooled animals at each time point. Figure shows a representative experiment out of three performed.

The expression of IL-17A was analyzed in total lung single cell suspensions along the course of sub-lethal infection. Lymphocyte Gate was determined by using physical parameters (forward and side scatters). The expression of IL-17A was mostly below detection threshold in the non-lymphocyte cells (determined as all of the cells that were not included in the Lymphocyte Gate) throughout the course of infection (Figure 6A). IL-17A was detected only in some cells within the Lymphocyte Gate, an expression that increased during the course of infection (Figure 6A). Thus, these results indicate that the IL-17A detected in BALF may be mostly attributed to lymphocytes.

To evaluate the contribution of the various pulmonary T-cell populations to the net production of IL-17A, the actual number of the relevant cells (CD4^+^ and γδ T cells) was determined in the course of infection. The basal absolute amount, prior to infection, of the CD4^+^ T cells was found to be >100-fold higher than that of γδ T cells (Figure 6, B–C). Cell number analysis along sub-lethal infection showed that the amount of pulmonary γδ T cells has peaked dramatically by days 5-6 and subsided subsequently (Figure 6B). In contrast, CD4^+^ T cells exhibited a persistent gradual increase in absolute numbers, starting from day 6 and on (Figure 6C). This kinetics underlines the innate-like properties of γδ T cells, as compared to the adaptive-like kinetics of CD4^+^ T cells. Nevertheless, there were still at least 10-fold more CD4^+^ T cells than γδ T cells throughout the course of disease (compare Figure 6B and 6C).

The actual numbers of IL-17A producing cells in these two T cell populations were extrapolated. Results suggest that CD4^+^ cells are the major contributors to IL-17A production in infected LVS lungs, yet γδ T cells may have a role in the initial phase of infection (Figure 6D). This is underlined when the representation of each subpopulation within the IL-17A-producing cells is calculated (Figure 6E). In accordance with the rapid kinetics of γδ T cells, the amount of IL-17A^+^γδ T cells was comparable to that of the CD4^+^IL-17A^+^ cells until Day 5, comprising 25–40% of the total IL-17A^+^ cells (Figure 6E). From day 6 and on the proportion of IL-17A^+^ γδ T cells has consistently declined down, as Th17 cells became the dominant source for IL-17A (Figure 6E).

In line with previous observations, NK cells displayed an early and rapid induction in the production of IFNγ, which peaked on day 8 and then declined. IL-4, IL-5 and IL-17A were not expressed by NK cells at all (data not shown).

### γδ T cells are not essential for survival from intranasal LVS infection

**Figure 7:**
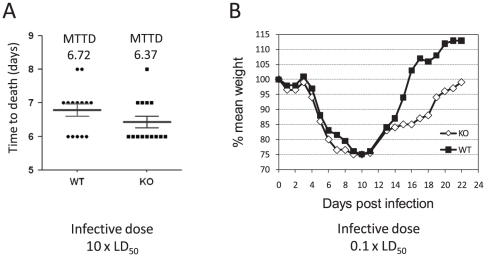
The effect of γδ T cells knockout on response to intranasal LVS infection. Wild type (WT) or TCRδ^−/−^ (KO) C57BL/6 mice were intranasally infected with a lethal 10×LD_50_ (A) or a sub-lethal 0.1×LD_50_ (B) infective dose. (A) compares the mean time to death (MTTD) and (B) compares the mean percentage of body weight monitoring of the WT and KO groups.

Since γδ T cells were identified as an early source for IL-17A during LVS infection (Figure 6), their role in the response to LVS was tested. TCRδ^−/−^ (KO) and wild type (WT) C57BL/6 mice were infected either with a lethal dose of 10×LD_50_ or with a sub-lethal dose of. Although the mean time to death of TCRδ^−/−^ mice infected with 10×LD_50_ was slightly lower than that of the WT mice (6.37 days vs. 6.72 days), this difference was not statistically significant (Figure 7A).

Infection with a dose of 0.1×LD_50_, which is sub-lethal to WT mice, proved to be sub-lethal to the KO mice as well, suggesting that γδ T cells are not essential for mice recovery from infection. Moreover, following sub-lethal infection, TCRδ^−/−^ mice displayed a similar course of disease to WT mice, except for the period of convalescence (Figure 7B). Indeed, convalescence period was significantly prolonged in TCRδ^−/−^ as compared to WT, with a lag of at least 7 days in reaching basal body weight (Figure 7B). These results indicate that γδ T cells play only a partial role in the response to LVS, which is not essential for survival.

### Exogenous administration of IL-17A moderately delays time of death from lethal intranasal LVS infection

**Figure 8:**
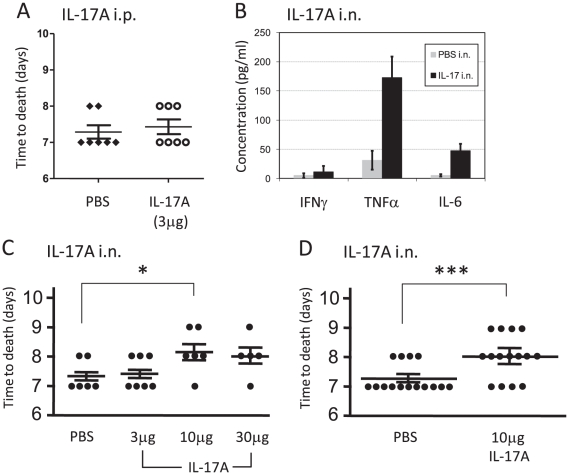
The effect of IL-17A administration on response to intranasal LVS infection. WT C57BL/6 mice were infected intranasally with a lethal dose of 10×LD_50_. (A) shows the effects on mice survival of intraperitoneal systemic administration of IL-17A or carrier only at days 0 and 3 post infection with 10×LD_50_. The time of death of each animal is shown; (B) depicts the effect of intranasal administration of IL-17A in naïve mice on cytokine production. 24 h after instillation of IL-17A (black bars) or PBS carrier (gray bars), BALFs were harvested from three individual animals and the concentrations of the indicated cytokines were determined. Results show the mean concentration values; (C) shows the effects of intranasal administration of IL-17A or carrier only at days 0 and 3 post-infection with 10×LD_50_. Three doses of IL-17A were tested, as indicated in the figure. The time of death of each animal is shown. Asterisks represent a statistically significant difference (P value <0.05). Results of one experiment out of two performed is presented; (D) shows the pooled results of three independent experiments performed only with the dose of 10 µg IL-17A versus carrier only. Each experimental mice group in each independent experiment included five mice.

The potential beneficial effect of exogenous administration of IL-17A was tested in a model of lethal infection with 10×LD_50_. Systemic administration of 3 µg of IL-17A (intraperitoneal injection) on the day of infection and on day 3 did not provide any benefit when compared to the control PBS treatment (Figure 8A). Similar results were obtained when IL-17A was administered one day before the infection (data not shown). We therefore hypothesized that IL-17A should be administered directly to the target organ of infection, the respiratory tract. Intranasal instillation of IL-17A resulted in enhanced concentrations of TNFα and IL-6 in the BALF, but not of IFNγ, one day after instillation (Figure 8B). This concurs with known biological activities of IL-17A [26].

The effect of intranasal instillation of IL-17A (at days 0 and 3) was tested in escalating doses: 3 µg, 10 µg and 30 µg per mouse, as compared to instillation of the carrier only (PBS). Instillation of 3 µg did not provide any benefit as compared to control PBS (Figure 8C). The mean time to death was increased mildly, yet in a statistically significant manner, by 0.7 days when 10 µg of IL-17A were administered. This benefit was reproduced in several independent experiments (Figure 8D). A similar effect was observed with 30 µg, which was not superior to administration of 10 µg of IL-17A (Figure 8C). Thus, the mild beneficial effect cannot be further improved by higher doses of exogenous IL-17A.

### Exogenous administration of IL-23 moderately delays time of death from lethal intranasal LVS infection

**Figure 9:**
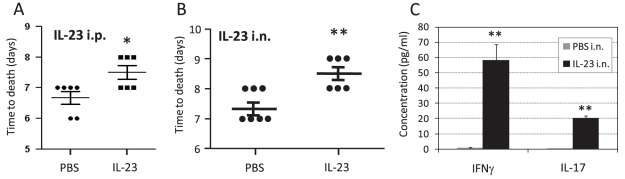
The effect of IL-23 administration on response to intranasal LVS infection. WT C57BL/6 mice were infected intranasally with a lethal dose of 10×LD_50_. (A) shows the effects on mice survival of systemic intraperitoneal administration of IL-23 or carrier only at the day of infection with 10×LD_50_. The time of death of each animal is shown; (B) shows the results of intranasal administration of IL-23 or carrier only at the day of infection with 10×LD_50_. (C) depicts the effects of intranasal administration of IL-23 in naïve mice. 24 h after instillation of IL-23 (black bars) or PBS carrier (gray bars), BALFs were harvested from three individual animals and the concentrations of selected cytokines were determined. Results show the mean concentration values; * represents statistical significance of P value <0.05, ** represents statistical significance of P value <0.01. Figure shows the result of a representative experiment out of three performed. In the other experiments, each mice group included 5-6 mice.

The cytokine IL-23 acts upstream to IL-17 in the IL-17 axis, and it promotes differentiation of Th17 cells and secretion of IL-17A [26]. We have therefore tested the effect of exogenous administration of IL-23 in the model of lethal infection with 10×LD_50_. As opposed to IL-17A, even a single systemic administration of 3 µg of IL-23 (intraperitoneal injection) on the day of infection provided a moderate, yet statistically significant, benefit when compared to the control PBS treatment. The mean time to death was increased by almost 1 day (Figure 9A). A similar 1-day delay in the onset of death was also observed when 3 µg of IL-23 were administered intranasally at the day of infection, as compared to control PBS instillation (Figure 9B). The beneficial effect of IL-23 could be accounted for by a combined induction of IL-17A and IFNγ, since instillation of IL-23 to naïve mice induced the production of both cytokines (Figure 9C). In conclusion, administration of IL-23 yielded only a partial effect, which was similar to the effect of exogenous IL-17A.

### IL-17A is essential to the response against sub-lethal intranasal LVS infection

In order to directly test the *in vivo* role of IL-17A in the response to sub-lethal LVS infection, IL-17A was depleted *in vivo* using anti-IL17A antibodies. The efficacy of IL-17A depletion was determined by measuring IL-17A in the BALF of mice 6 days post intranasal infection with 0.1×LD_50_ and intraperitoneal administration of the neutralizing antibody, or of PBS as control. At this time point, the IL-17A concentration in the BALF of PBS-treated animals was around 50 pg/ml, as compared to below detection level in the mAb-treated animals (data not shown).

**Figure 10:**
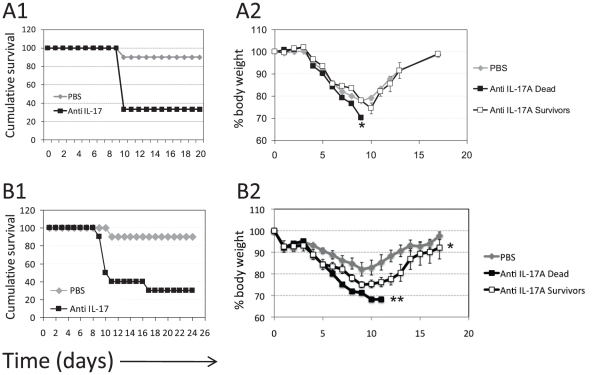
The effect of anti IL-17A antibody administration on response to intranasal LVS infection. (A) WT C57BL/6 mice were infected with 100 CFU (0.1×LD_50_). Mice were then injected with 100 µg of neutralizing anti IL-17A mAb (n = 6 animals) or with carrier only (n = 10 animals) on days 0 and 7 post infection. (A1) depicts the cumulative survival of the two experimental groups, P value <0.05; (A2) depicts body weight monitoring of PBS-treated mice (gray diamonds), mAb-treated mice that eventually died (black squares, n = 4 animals) or recovered of the disease (white squares, n = 2 animals). Figure shows one experiment out of two performed (with similar results); (B) WT C57BL/6 mice were infected with 30 CFU. Mice were then injected with 100 µg of neutralizing anti IL-17A mAb (n = 10 animals) or with carrier only (n = 10 animals) on days 0 and 7 post infection. (B1) depicts the cumulative survival of the two experimental groups, P value <0.01; (B2) depicts body weight monitoring of PBS-treated mice (gray diamonds), mAb-treated mice that eventually died (black squares, n = 7 animals) or recovered of the disease (white squares, n = 3 animals). Figure shows one experiment out of two performed (with similar results). *denotes P value <0.05; ** denotes P value <0.01.

Next, mice were infected with 100 CFU (0.1×LD_50_) and treated with neutralizing anti IL-17A antibodies or PBS on the day of infection, and again seven days post-infection. In the PBS-treated group, all mice except of one successfully recovered from the disease and survived. Namely, five mice infected with 0.1×LD_50_ LVS treated with rat IgG2a isotype control did not succumb to the infection and exhibited a morbidity pattern similar to that of the PBS-treated mice (data not shown). Strikingly, treatment of mice with the neutralizing anti IL-17A led to a substantially decreased survival, as 66.6% of the infected mice died of infection, all on day 10 (Figure 10 A1). In terms of morbidity, monitored by body weight, the surviving mAb-treated mice exhibited a generally similar pattern of weight loss to that of PBS-treated mice (Figure 10 A2). However, the mAb-treated mice that eventually died on day 10 exhibited an accelerated course of disease.

Similar results were obtained in an experiment where mice were infected with 30 CFU. In the PBS-treated group, all mice except of one, successfully recovered and survived. The treatment with anti IL-17A antibodies ultimately resulted in the death of 70% of the mice (Figure 10 B1-B2). In this experimental setup, the effect of IL-17A depletion on morbidity was clearly manifested. Among the mAb-treated group, the mice that eventually died demonstrated a sharp drop in body weight evident from day 6 post-infection. Moreover, the effect of antibody treatment on morbidity could also be observed in the surviving mice (nadir of 75%), as compared to the PBS-treated mice (nadir of 82%) (Figure 10 B2).

Treatment of mice with neutralizing anti-IL17 antibodies accelerated also the course of disease in mice infected with a lethal dose of 5×LD_50_. The mean time to death of PBS-treated mice was 9.1 days and of the anti-IL17 treated mice was 7.8 days (p value <0.05, data not shown). Treatment with anti IL-17A antibodies did not induce by itself any toxic effects in naïve mice (data not shown).

In conclusion, these results show that IL-17A plays a pivotal role in the immune response against intranasal LVS infection.

## Discussion

One of the most severe forms of tularemia is *inhalational tularemia*, which directed the present work towards studying components of the pulmonary immune system in the response to airway LVS infection. We have utilized an *in vivo* murine model of sub-lethal inhalational LVS infection, which causes a self limiting disease, in an attempt to identify critical protective determinants, which could lead to the development of novel preventive and interventional modalities.

Temporal overlay of bacterial counts and immune activation following this sub-lethal infection showed that on day 8 bacterial counts peaked, body weight was at the nadir, and pulmonary lymphocytes (T & NK cells) reached maximal activation, which was sustained thereafter (Figure 2). The immune activation plateau coincided with clearance of the bacteria locally and systemically, as well as with clinical convalescence (Figure 2). This would suggest that the cellular immune responses might be involved in mediating the clearance of LVS and eventual recovery.

The interrelationship of this intracellular bacterium with the immune system is under continuous investigations and appears to be complex. It involves recruitment of host anti-bacterial measures such neutrophils, macrophages, inflammatory response and most importantly Th1-mediated specific IFNγ and CTL responses [11], [16], [21], [24]. However, several bacterial immune evasion mechanisms have already been identified, including downregulation of CD14 and TLR expression, as well as downregulation of post receptorial signaling machineries [36]. In addition, *Ft* was shown to stimulate the production of suppressive mediators like TGFβ [7], [33] or Prostaglandin E_2_ [37].

Due to the temporal coincidence between pulmonary lymphocyte activation in the sub-lethally infected lungs, bacterial clearance and convalescence (Figure 2), we have screened the kinetics of mRNA production of an array of cytokines in the isolated pulmonary lymphocytes during infection. Surprisingly, while the expected upregulation of Th1 cytokines such as IFNγ was observed [11], [16], [21], [24], it was preceded by a strong and early induction of Th2 transcripts, such as IL-4, IL-5 and IL-13. In fact, it was previously reported that *Ft*-infected macrophages release Protaglandin E2 that promotes Th2-like responses [37]. In most cases, the effective immune response elicited against intracellular pathogens is Th1 cell-mediated immunity [38]–[39]. Indeed, it was previously reported that humoral antibody-mediated response is probably only minimally involved in the protection from LVS infection [6]. Thus, diversion of the elicited immune response by LVS bacteria towards humoral response could emerge as an immune evasion mechanism. It should be noted that LVS is a highly attenuated strain and the possible relevance of such mechanism should be investigated in the virulent SchuS4 strain. Noteworthy, nevertheless, is the observation that the early and prominent increase in Th2 transcripts was short-lived (Figure 3) and did not culminate in production of measurable cytokines *in vivo* (Figure 5). The observed termination of the early expression of Th2 cytokines could occur due to the concurrently elicited strong Th1 response. For example, early production of IL-12 by activated macrophages and massive secretion of IFNγ by NK cells could redirect the immune response towards Th1.

While it is well established that IFNγ response plays a central role in anti *Ft* response, a study on mice deficient of the IL-12p35 subunit suggested that other mechanisms could be involved in host response to LVS. These mice are unable to form a functional IL-12 complex, which hinders their ability to mount an IFNγ response. Yet, they were still able to be cleared of intradermal LVS infection [23]. An alternative mechanism that could be involved in protection from LVS could depend on the IL-17 axis. Notably, IL-12p40, which is still expressed in IL-12p35^−/−^ mice, can associate with the IL-23p19 subunit to form a functional IL-23 [40]. IL-23 promotes the IL-17 axis through differentiation of helper T cells into IL-17A-producing Th17 cells [41]. IL-17A is an early proinflammatory cytokine, promoting several effective host defensive mechanisms [reviewed in 26] against a broad spectrum of pathogens, including major intracellular bacteria such as *M. tuberculosis, M. pneumoniae and L. monocytogenes* [reviewed in 26]–[28], but it has been only poorly investigated so far in *Francisella* infections.

Here we show that IL-17A is produced in the lungs in response to airway LVS infection (Figure 4). This was demonstrated at the mRNA level, the overall cytokine level and the amount of IL-17A-producing lymphocytes (γδ T cells and Th17 cells) in the infected lungs (Figures 3–6). Noteworthy, the production of IL-17A following sub-lethal airway infection was of transient nature, peaking around days 4-6 and apparently preceding the full-blown disease (days 6–8). This profile could thus suggest that IL-17A is involved in restraining the infection and preventing it from becoming fulminant. However, as opposed to IFNγ and IL-6, the production of IL-17A could not be further induced by infection doses beyond 10×LD_50_, which could be explained by reaching the maximal production capacity by the host or, alternatively, by a bacterial inhibitory mechanism that hinders the production or affect the sustainability of IL-17A.

γδ T and CD4^+^ Th17 cells were identified as the main pulmonary lymphocyte sources for IL-17A following LVS airway infection (Figures 5–6). It should be noted that the gating approach was based on forward and side scatters, which is suboptimal (e.g. to FSC-A vs. FSC-W) when non-lymphocyte populations may be involved. Important roles in host response to various pathogens were previously demonstrated for both T cell subpopulations. The anti pathogen roles of γδ T cells, a unique subpopulation of T cells with invariant T cell receptor and innate-like properties, has gained much attention lately, especially due to their role in secretion of IL-17A [29]. It was very recently shown that the IL-17A-producing γδ T, but not other γδ T cells, express TLR1, TLR2 and dectin-1, respond to IL-23 and recognize various pathogens [42]. The role of γδ T cells in tularemia has been poorly studied so far, including mainly demonstrations of a substantial increase in the proportion of circulating γδ T cells in human tularemia patients [43]–[44]. Our results suggest that pulmonary γδ T cells are an early source for IL-17A (Figure 6) and that they exhibit rapid activation kinetics evident by CD69 expression (data not shown). Nevertheless, TCRδ^−/−^ knockout mice were not more vulnerable airway LVS infection (Figure 7), as they did not succumb to sub-lethal airway infection and the mean time to death due to lethal airway infection was not significantly different from the wild type mice. Noteworthy, it was previously reported that γδTCR^−^ mice do not succumb to sub-lethal intra-dermal LVS infection as well [17]. Interestingly, the airway infected knockout mice did exhibit a delayed recovery period from sub-lethal infection, as compared to the wild type mice. Thus, γδ T cells are not essential for mounting effective protective response to LVS, but conceivably they play some role in clearance of the pathogen.

Notably, exogenous administration of IL-17A during lethal airway infection yielded limited results. A mild beneficial effect was observed only following administration of high doses of recombinant IL-17A and it could not be further enhanced with higher doses (Figure 8). Exogenous administration of IL-23 yielded similarly limited benefits (Figure 9). The limited beneficial effects of exogenous cytokine administration in lethal airway infections could be explained by: a) failure to reach an adequate functional concentration in the target organs; b) IL-17A activity and stability could be altered in the microenvironment of LVS-infected respiratory tract; c) the existence of a putative counter mechanism of IL-17A exerted by the bacteria, which overcomes the administered cytokine; d) the limited ability of the host to adequately respond to IL-17A and develop a clinically significant effect may be physiologically limited.

While exogenous administration of IL-17A did not provide fully satisfying results, we directly show the protective role of IL-17A in anti-LVS host response by the *in vivo* IL-17A neutralization experiments (Figure 10). Indeed, *in vivo* neutralization of IL-17A with anti IL-17A monoclonal antibody caused the majority of the treated mice to develop a more severe disease and succumb to an otherwise sub-lethal dose (100 CFU or 30 CFU, Figure 10). Accordingly, the anti IL-17A shortened the time to death in a lethal infection setup of 5×LD_50_ (data not shown). The depletion of IL-17A also exacerbated disease manifestations, evident mainly following infection with 30 CFU (Figure 10). The exact mechanisms by which the IL-17A response contributes to host protection against LVS is still unclear, but could be mediated, for example, by neutrophil recruitment or induction of inflammatory cytokines such as IL-6, GM-CSF and TNFα [45]–[46]. The short-lived IL-17A response probably reflects the tight regulation on this immune effector arm, which can facilitate severe inflammatory responses and autoimmune manifestations [26]. Since we demonstrate that the presence of IL-17A is important for protection from LVS (Figure 10), but the IL-17A-secreting γδ T cells are non-essential (Figure 7), it could be speculated that other cellular sources of IL-17A, such as Th17 -cells (Figure 5), produce sufficient amounts of IL-17A to resolve the low-dose infection.

It was very recently published that IL-23-Th17 pathway regulates the IL-12-Th1 cell pathway and is required for protective immunity against *F. tularensis* live vaccine strain [34]. Most of the work in that report was performed with a series of knockout mice, including knockouts of IL-23, IL-17A and IL-17A-Receptor. *In vivo* depletion of IL-17A with an antibody impaired bacterial clearance from the lungs [34]. In another very recently published report, the protective role of IL-17A against intradermal LVS infection was demonstrated with IL-17A knockout mice, while effects of exogenous cytokines promoting the IL-23/IL-17A axis were demonstrated *in vitro* in an intracellular LVS growth system [35].

Here we provide comprehensive accurate data on the kinetics of the IL-17-mediated response, including the production of IL-17A in the lungs and the identity of the lymphocyte sources for IL-17A, with regard to sub-lethal and lethal infective doses. Furthermore, we show that *in vivo* modulation of the IL-23/IL-17A axis either by administration of exogenous cytokines (IL-17A or IL-23) or IL-17A depleting antibody, does not only result in immune modulation or altered bacterial clearance, but also directly affect resilience of animals to intranasal LVS infection, evident by clinical endpoints such as morbidity patterns (reflected by body weight) and cumulative survival.

Taken together, the accumulating evidence indicates that the IL-23/IL-17A axis plays a role in the protection of the infected host against F. *tularensis*.

## Materials and Methods

### Ethics statement

All experiments reported here were conducted in compliance with the guidelines of the animal use committee at the Israel Institute for Biological Research and are in accordance with the Animal Welfare Act.

### Antibodies and cytokines

The following fluorochrome-conjugated monoclonal antibodies, which were all purchased from eBioscience were used in this work (the number in brackets represents the titrated working concentration): PE-conjugated anti-Thy1.2 (0.05 µg/10^6^ cells), PE-Cy5.5-anti mouse CD3 (0.1 µg/10^6^ cells), FITC-anti mouse αβ TCR (0.05 µg/10^6^ cells), FITC-anti mouse CD4 (0.1 µg/10^6^ cells), APC-anti mouse CD8 (0.05 µg/10^6^ cells), APC-anti mouse NK1.1 (0.15 µg/10^6^ cells), FITC-anti mouse DX5 (0.15 µg/10^6^ cells), PE-anti mouse CD69 (0.1 µg/10^6^ cells), PE-anti mouse CD25 (0.1 µg/10^6^ cells), PE-anti mouse IFNγ (0.1 µg/10^6^ cells), PE-Cy5.5 anti mouse IL-4 (0.1 µg/10^6^ cells), PE-Cy5.5 anti mouse IL-5 (0.125 µg/10^6^ cells), PE-anti mouse IL-17A (0.125 µg/10^6^ cells), APC-anti mouse γδ TCR (0.1 µg/10^6^ cells). All appropriate fluorochrome-conjugated isotype controls were purchased from eBioscience. In addition, the neutralizing anti mouse IL-17A and the rat IgG2a isotype control were used (R&D System, Minneapolis MN USA, clone 50104). The anti IL-17A antibodies (for flow cytometry and for *in vivo* neutralization) are specific to this isoform and do not cross react with other isoforms. Recombinant murine IL-17A was purchased from Prospec (Rehovot, Israel) and recombinant murine IL-23 was purchased from R&D Systems.

### Animal studies

The main mouse strain used in this study was C57BL/6 (Harlan, Israel). Other strains used were the TCRδ^−/−^ mice (Jackson laboratories) and their C57BL/6 background (Jackson laboratories), as well as Balb/c mice (Harlan, Israel). Intranasal instillation to anesthetized mice was performed in a volume of 25 µl. Anesthesia was performed by intraperitoneal injection of Ketamine and Xylasine diluted in sterile PBS. In experimental protocols in which mice were infected and treated at the day of infection intranasally, both instillations were performed under a single anesthesia. Mice were first instilled with the treatment and after several minutes with the bacteria. Systemic administration of cytokines or antibodies was performed by intraperitoneal injection of the appropriate constituent diluted in 0.5 ml. Harvesting of bronchoalveolar lavage fluids (BALF) was performed in deeply anesthetized mice following previously reported methodology [9]. Briefly, the trachea was surgically exposed and cannulated with a 23g plastic catheter. The inserted catheter was further fixed in place with a 3-0 silk tie. Using a 1 ml syringe, 1 ml of PBS was pushed through the catheter and immediately collected back. In almost all cases, 700 µl of BALF were obtained. In rare cases of blood contamination, the samples were excluded and discarded.

### Tissue processing

Various organs, including spleen, lungs and mediastinal lymph nodes (MdLN) were harvested from euthanized mice. Organs were rendered into single cell suspensions by mechanical and/or enzymatic digestion as previously described [9]. Briefly, spleens were gently crushed with a 3 ml syringe (BD Biosciences), gently pipetted and passed through cell strainer (BD Biosciences) to eliminate aggregates. Lungs were washed from external blood and minced into 1–2 mm pieces. The minced pieces were further incubated for 1 h with Liberase Blendzyme 3 (Roche) in a final concentration of 2 µg/ml at 37 degrees Celsius, followed by 5 minute treatment with 100 U/ml of DNAse I (Roche) in 37 degrees Celsius. Mediastinal lymph nodes were subjected only to enzymatic digestion, exactly as described above. After the incubation with the enzymes, suspension was thoroughly pipetted until a single cell suspension was obtained. The suspension was then passed through a cell strainer.

### Bacterial preparation and enumeration

*Francisella tularensis* live vaccine strain (ATCC 29684) stocks were plated on CHA agar (GC Medium base, Difco, supplemented with 1% hemoglobin and 1% Iso-Vitalex BD, France). Working stocks were prepared from single individual colonies exhibiting the large-light phenotype [47]. For cell and animal infection experiments, bacteria were grown at 37 degrees Celsius to mid log phase (optical density of 0.1–0.2 at 660 nm) in TSBC (TSB Difco, supplemented with 0.1% cysteine) in a gyrostatory shaker. Bacteria were washed and then re-suspended at the desired concentration in PBS for animal infection experiments. The intranasal LD_50_ of our LVS bacterial stocks was rigorously determined and defined as 10^3^ CFU for the C57BL/6 mice [9], which was similar to reports by others [32]. Bacterial enumeration in various organs was performed by serial dilutions of single cell suspensions, plating on CHA for 48–72 hours in 37 degrees Celsius and counting of formed colonies. Killed bacterial suspensions were generated by incubating log phase bacterial cultures in the presence of 0.4% formaldehyde overnight at room temperature, followed by extensive washing with PBS.

### Flow cytometry

Single cell suspensions derived from spleen, lungs or MdLN were centrifuged at 500g for 5 minutes. Red blood cells were lyzed using Red Cell Lysis solution (Sigma-Aldrich, Israel) according to manufacturer's instructions and washed with FACS buffer (PBS/BSA 0.5%/Sodium-Azide 0.02%). Fc receptors were blocked by incubation of 1×10^6^ cells with 1% Fc Block (Miltenyi, Germany) diluted in 50 µl FACS buffer, for 15 minutes on ice. Cells were then plated in 96-U microplates (Nunc). The appropriate antibody mixes or single stains in the optimized concentrations (diluted in FACS buffer) were then added onto the Fc blocked cells in additional 50 µl and incubated for 30 minutes on ice in dark conditions. The plate was then centrifuged at 500 g for 5 minutes in 4 degrees Celsius, supernatant was removed and each well was further washed by 200 µl of FACS buffer. After the wash, cells were transferred to FACS acquisition tubes through a mesh to eliminate aggregates for analysis in FACScalibur instrument and CellQuest software. The lymphocyte subpopulation was gated according to standard Forward and Side Scatter parameters, in order to avoid irrelevant larger cell populations or smaller cell populations, fragments and debris. A low proportion of dead cells (<10%) was verified by PI staining. When possible, PI was used in the same test tube with the antibodies, depending on the fluorescence channels occupied by the antibodies.

### Intracellular cytokine staining

This approach is based on the assumption that the pulmonary lymphocytes isolated from infected lungs were already stimulated *in vivo*. The isolated pulmonary lymphocytes cells were pre-incubated in 37 degrees Celsius for 4 hours with Brefeldin A (eBioscience) diluted in complete medium, comprised of RPMI-1640 (Biological Industries, Bet Haemek, Israel) supplemented with 10% heat inactivated fetal calf serum (Biological Industries, Bet Haemek, Israel) and 1 mM of Pen-Strep (Biological Industries, Bet Haemek, Israel), non essential amino acids (Biological Industries, Bet Haemek, Israel), L-glutamine (Biological Industries, Bet Haemek, Israel) and sodium pyruvate (Biological Industries, Bet Haemek, Israel). The cells were not incubated with any exogenous stimulant. Pen-Strep was added to eliminate residual bacteria that were carried over from the in vivo infection. Next the cells were collected, centrifuged and washed with FACS buffer. After the cells were stained extracellularly as described above, cells were fixed and permeabilized by using the cytofix/perm and perm/wash solutions (eBioscience) according to the manufacturer's instructions. Then, the appropriate antibody mixes or single stains diluted in permeabilization solution in the recommended concentrations were added for 30 minute incubation on ice under dark conditions. Cells were then washed and transferred to FACS acquisition tubes for analysis in FACScalibur instrument and CellQuest software.

### Cell sorting of pulmonary lymphocyte subpopulations

Pulmonary single cell suspensions from at least 7 individual C57BL/6 animals were pooled together and underwent Red Cell Lysis (Sigma-Aldrich, Israel) according to manufacturer's instructions. Lymphocytes were enriched by incubation in complete medium (described above) in sterile tissue culture 10 cm plates (Falcon, USA) for overnight in 37 degrees Celsius to allow adherence of cells. Non-adherent cells were gently collected, washed and passed through a cell strainer. The cells were extracellularly stained with FITC-anti DX5 and PE-Cy5.5-anti CD3. Staining was performed as described above with the exception that RPMI-1640 medium with 0.5% FCS was used and not standard FACS buffer. Cells were transferred into sorting tubes in a concentration of 1×10^7^ cells/ml diluted in RPMI. Cells were sorted using FACSvantage instrument into NK cells and T cells by applying gates on appropriate physical (Forward and Side Scatters typical for lymphocytes) and fluorescent characteristics. NK cells were defined as DX5^+^CD3^−^ cells and T cells were defined as DX5^−^CD3^+^ cells within the lymphocytes' gate. In each sorting session, 5×10^5^–1×10^6^ cells of each desired subpopulation were collected. The acquisition and collection tubes were kept in ice throughout the entire process. The purity of each sorted subpopulation was >95% as validated by post-sorting staining with PE-anti Thy1.2 and APC-anti NK1.1. Cells were sorted from naïve animals, as well as from certain time points after infection with 0.1×LD_50_.

### ELISA

Quantification of murine TNFα, IL-6, IFNγ and IL-17A was performed by using Duo-Set ELISA kits (R&D Systems) according to manufacturer's instructions. The kit for IL-17A is specific to this isoform and does not cross react with other isoforms.

### Real time PCR

Total RNA was purified by using RNAeasy kit (Qiagen, Germany) and converted to cDNA using OmniScript reverse transcriptase (Qiagen, Germany) according to manufacturer's instructions. For real-time PCR, 100 ng cDNA was amplified in 50 µl reaction using 500 nM primers, 5 mM Magnesium, 0.2 mM dNTP, PCR buffer, 100 nM Super ROX, AmpliTaq Gold DNA polymerase and EVA Green. Specific primers allowed quantification of the cytokines IL-1β, IL-2, IL-3, IL-4, IL-5, IL-6, IL-7, IL-10, IL-12p40, IL-13, IL-15, IL-17A, IL-18, IFNγ, TNFα, TGFβ, MIC-1 and GM-CSF. The sequences of the primers were published previously [48]. Experiments were done in triplicates and analyzed using the 7500 ABI Real Time PCR System (Applied Biosystems, USA). Analysis was performed as followed: for each cell sample the Ct value of GAPDH (the normalizing house keep gene used) was subtracted from the Ct value of each specific cytokine to obtain the ΔCt value (Ct_cytokine_-Ct_GAPDH_ = ΔCt). The ΔCt values in cells derived from naïve mice were used as reference values to get the ΔΔCt value (ΔCtx_infected_-ΔCtx_naive_ = ΔΔCt) and the 2^−ΔΔCt^ ratio (fold of change, relative to naïve cells).

## Supporting Information

Figure S1 (A) CD69 expression analysis on gated NK cells (NK1.1+CD3-TCRab- cells) in the lungs (closed diamonds) and MdLN (open squares) at the indicated time points after infection (B) CD69 expression analysis on gated T cells (NK1.1-CD3+TCRab+ cells). (C) and (D) show the total numbers of NK or T cells, as defined above; Cells were pooled from three animals at each time point and analyzed; Figure shows a representative experiment out of three independent experiments performed. Each time point included three animals.
(1.40 MB EPS)

Figure S2 (A) CD69 expression analysis on gated NK cells (NK1.1+CD3-TCRab- cells) in the lungs (closed diamonds) and MdLN (open squares) at the indicated time points after infection (B) CD69 expression analysis on gated T cells (NK1.1-CD3+TCRab+ cells). (C) and (D) show the total numbers of NK or T cells, as defined above; Cells were pooled from three animals at each time point and analyzed; Figure shows a representative experiment out of three independent experiments performed. Each time point included three animals.
(1.59 MB EPS)
